# PD-L1 Is Not Constitutively Expressed on Tasmanian Devil Facial Tumor Cells but Is Strongly Upregulated in Response to IFN-γ and Can Be Expressed in the Tumor Microenvironment

**DOI:** 10.3389/fimmu.2016.00581

**Published:** 2016-12-09

**Authors:** Andrew S. Flies, A. Bruce Lyons, Lynn M. Corcoran, Anthony T. Papenfuss, James M. Murphy, Graeme W. Knowles, Gregory M. Woods, John D. Hayball

**Affiliations:** ^1^Menzies Institute for Medical Research, University of Tasmania, Hobart, TAS, Australia; ^2^Experimental Therapeutics Laboratory, Hanson Institute, School of Pharmacy and Medical Science, University of South Australia, Adelaide, SA, Australia; ^3^Experimental Therapeutics Laboratory, Sansom Institute, School of Pharmacy and Medical Science, University of South Australia, Adelaide, SA, Australia; ^4^School of Medicine, University of Tasmania, Hobart, TAS, Australia; ^5^Walter and Eliza Hall Institute of Medical Research, Parkville, VIC, Australia; ^6^Department of Medical Biology, The University of Melbourne, Melbourne, VIC, Australia; ^7^Computational Cancer Biology Program, Peter MacCallum Cancer Centre, Melbourne, VIC, Australia; ^8^Mount Pleasant Laboratories, Tasmanian Department of Primary Industries, Parks, Water and the Environment, Prospect, TAS, Australia; ^9^Discipline of Obstetrics and Gynaecology, School of Medicine, Robinson Research Institute, The University of Adelaide, Adelaide, SA, Australia

**Keywords:** transmissible tumor, inhibitory checkpoint molecule, DFTD, wild immunity, PD-1, PD-L1, marsupial, allograft

## Abstract

The devil facial tumor disease (DFTD) is caused by clonal transmissible cancers that have led to a catastrophic decline in the wild Tasmanian devil (*Sarcophilus harrisii*) population. The first transmissible tumor, now termed devil facial tumor 1 (DFT1), was first discovered in 1996 and has been continually transmitted to new hosts for at least 20 years. In 2015, a second transmissible cancer [devil facial tumor 2 (DFT2)] was discovered in wild devils, and the DFT2 is genetically distinct and independent from the DFT1. Despite the estimated 136,559 base pair substitutions and 14,647 insertions/deletions in the DFT1 genome as compared to two normal devil reference genomes, the allograft tumors are not rejected by the host immune system. Additionally, genome sequencing of two sub-strains of DFT1 detected greater than 15,000 single-base substitutions that were found in only one of the DFT1 sub-strains, demonstrating the transmissible tumors are evolving and that generation of neoantigens is likely ongoing. Recent evidence in human clinical trials suggests that blocking PD-1:PD-L1 interactions promotes antitumor immune responses and is most effective in cancers with a high number of mutations. We hypothesized that DFTD cells could exploit the PD-1:PD-L1 inhibitory pathway to evade antitumor immune responses. We developed recombinant proteins and monoclonal antibodies (mAbs) to provide the first demonstration that PD-1 binds to both PD-L1 and PD-L2 in a non-placental mammal and show that PD-L1 is upregulated in DFTD cells in response to IFN-γ. Immunohistochemistry showed that PD-L1 is rarely expressed in primary tumor masses, but low numbers of PD-L1^+^ non-tumor cells were detected in the microenvironment of several metastatic tumors. Importantly, *in vitro* testing suggests that PD-1 binding to PD-L1 and PD-L2 can be blocked by mAbs, which could be critical to understanding how the DFT allografts evade the immune system.

## Introduction

In 1996, a clonal, transmissible tumor was identified in wild Tasmanian devils, *Sarcophilus harrisii* (1), in northeastern Tasmania and has since spread across Tasmania. This devil facial tumor disease (DFTD) has been a primary cause of a devastating decline in the wild Tasmanian devil population (2). In 2015, a second transmissible tumor [devil facial tumor 2 (DFT2)] was discovered in wild devils and will undoubtedly hamper conservation efforts for the iconic species (3). Extensive monitoring of the wild population has shown that the devil facial tumor 1 (DFT1) is nearly always fatal once established in a host, and DFT2 appears to have similar lethal effects. A successful breeding program has led to the establishment of an insurance population of Tasmanian devils, but release of devils from the insurance population into the wild may prove futile until a vaccine that can protect against the DFTD is developed.

One of the initial hypotheses offered to explain the transmissible nature of the DFT1 was that the low genetic diversity of the island population of Tasmanian devils allowed the transmissible tumors to be viewed as self, rather than foreign, by the host immune system (4, 5). However, the genome of two DFT1 cell lines that were thoroughly analyzed had 136,559 base pair substitutions and 14,647 insertions/deletions as compared with two normal devil reference genomes. Furthermore, several studies have now demonstrated that the devils do contain sufficient genetic diversity to mount strong immune responses to foreign tissue (6, 7), including the ability to reject skin allografts but not reject skin autografts (8). Genome sequencing of two sub-strains of DFT1 detected greater than 15,000 single-base substitutions that were found in only one of the DFT1 sub-strains (9), demonstrating that the transmissible tumors are evolving.

Analysis of human cancers has led Blank et al. (10) to suggest that “foreignness can likely be guaranteed for cancers with high mutational loads,” which leads to a relative abundance of neoantigens. The genetic mismatch due to host–tumor differences and ongoing generation of somatic mutations in the transmissible tumors should provide suitable neoantigen targets for host antitumor responses, yet the devil immune system fails to reject both DFT1 and DFT2 cells. A primary means of immune evasion by the DFT1 is *via* downregulation of genes associated with antigen processing and presentation, such as β2-microglobulin (B2m) (11, 12). As a result, MHC class I (MHC I) is not expressed on the surface of DFT1 cells, but cell surface expression of MHC I can be upregulated by IFN-γ (11). One potential downside to IFN-γ stimulation is that powerful inhibitory cell surface signaling molecules, such as PD-L1, are also upregulated in response to IFN-γ signaling in a wide variety of human, mouse, and canine tumors (13–15). Indeed, the correlation between PD-L1 and IFN-γ was 100% in human melanocytic lesions (16). PD-L1 can also be upregulated by IFNα/β, GM-CSF, IL-4, IL-10, and VEGF in mice and humans [reviewed in Ref. (17)] on a variety of cell types, including epithelial cells, endothelial cells, myeloid DCs, tumor-associated macrophages, and myeloid-derived suppressor cells [reviewed in Ref. (13)], all of which are capable of inhibiting antitumor responses (18, 19).

Immunotherapy that blocks checkpoint molecule interactions, such as the PD-1:PD-L1 interaction, has achieved 10–87% response rates in a broad range of late-stage human cancers (20–22). Recent evidence suggests that blocking PD-1:PD-L1 signaling promotes antitumor responses by releasing preexisting CD8 T cells from inhibitory signaling between PD-1 and PD-L1 [reviewed by Tumeh et al. (23) and Rizvi et al. (24)]. Additionally, Le et al. (25) have recently shown that tumors with extensive somatic mutation are more susceptible to immune checkpoint blockade immunotherapy, where the high mutation group (1,782 mutations on average) had a 40% response rate and the low mutation group (73 mutations on average) had no objective responses to PD-1 blockade colorectal cancer treatment. Additionally, PD-1 signaling has been shown to play a key role in graft survival in mice (26), thus making the DFTs, which are essentially tissue allografts, an attractive target for PD-1:PD-L1 immunotherapy.

The lack of devil-specific antibodies for flow cytometry and immunohistochemistry (IHC) has been a major obstacle in understanding the role PD-1:PD-L1 signaling, and immune function in general in devils. Here, we have developed a panel of α-PD-1 and α-PD-L1 monoclonal antibodies (mAbs) that are highly specific for devil proteins and are capable of blocking PD-1 binding to PD-L1 and PD-L2. We used these new mAbs to show that PD-L1 is strongly upregulated on DFT1 and DFT2 by IFN-γ *in vitro* and that PD-L1 was detected in the microenvironment DFT1 *via* IHC.

## Materials and Methods

### Tasmanian Devil Tissue Samples

Tasmanian devil PBMCs, tissues, and cell lines were collected with appropriate approvals from the Tasmanian Devil Captive Research Advisory Group, Tasmanian Government Department of Primary Industries, Parks, Water and the Environment (DPIPWE), Animal Ethics Committees from the University of South Australia (# U18-14), and the University of Tasmania (A0012513 and A0014976) and were performed in accordance with relevant guidelines and regulations. PBMCs were isolated by diluting 1:1 with PBS, layering on Histopaque at room temperature, and centrifuging at 400 g for 30 min. The PBMC layer was then collected and washed 2× with RPMI at 500 g for 5 min. Formalin-fixed paraffin-embedded (FFPE) tissue sections were collected and processed as reported previously (3, 27).

### Cells and Cell Culture Conditions

Devil facial tumor 1 cell lines C5065, 1426, and 4096, the DFT2 cell line JV, and devil PBMCs were cultured at 35°C in 5% CO_2_ in complete RPMI supplemented with 10% FBS (cRF10), 2 mM l-glutamine, penicillin–streptomycin (100 U/ml and 0.1 mg/ml), 10 mM HEPES, and 0.5 mM β-mercaptoethanol. Chinese hamster ovary (CHO) cells were cultured at 35°C with 5% CO_2_ in 5% complete RPMI supplemented with 5% FBS (cRF5). For production of recombinant proteins, stably transfected CHO cells were cultured in suspension in spinner flasks in chemically defined, serum-free CHO Ex-Cell (Sigma # 14361C) media supplemented with l-glutamine and penicillin–streptomycin.

### Identification and Analysis of Devil PD-1, PD-L1, PD-L2, and IFN-γ Genes

**Figure 1:**
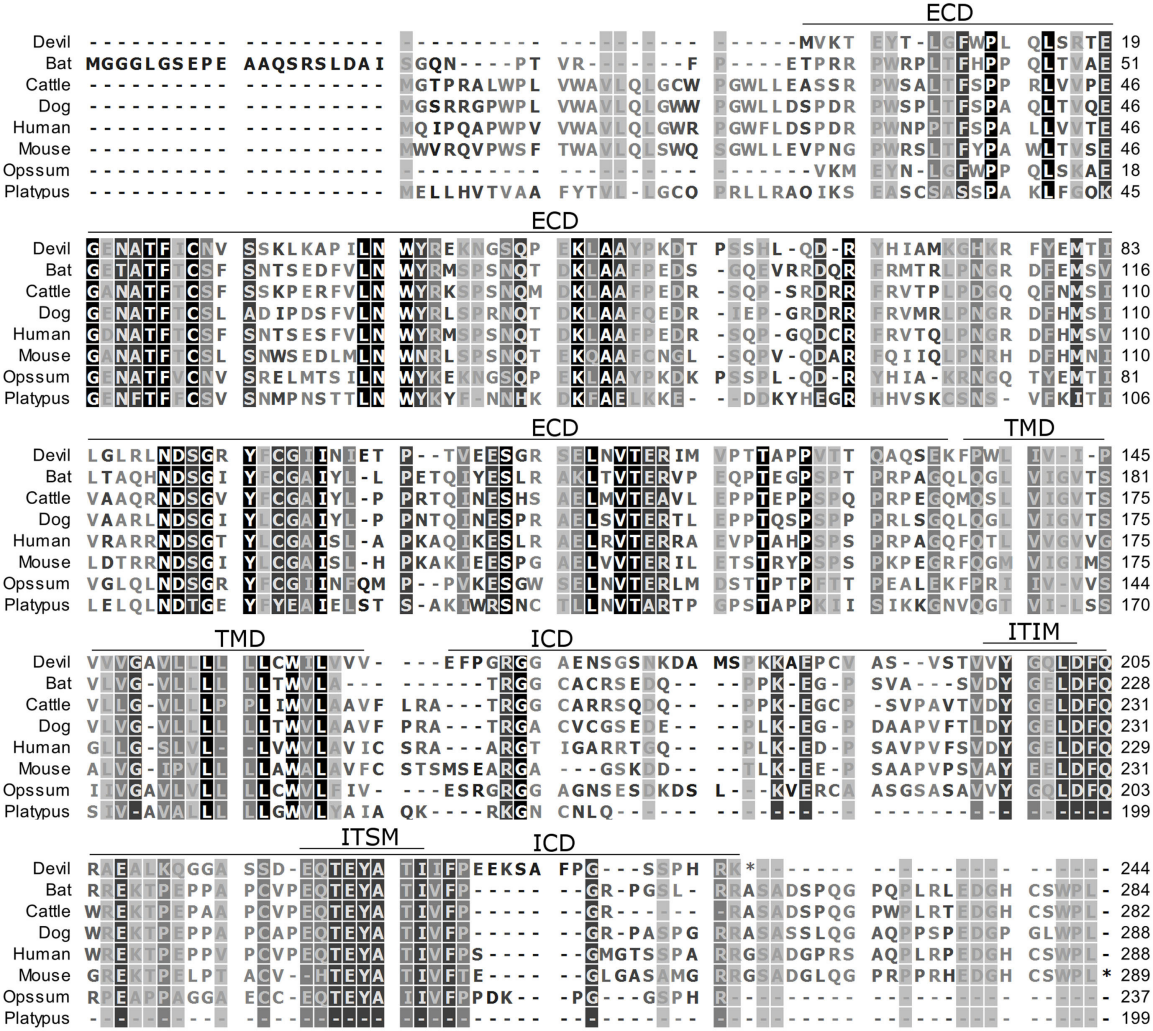
**Alignment of PD-1 amino acid sequence in eight mammalian species**. The black-to-white color gradient indicates the degree of amino acid conservation among species, with black boxes indicating the most highly conserved sequence regions and white boxes indicating the most divergent regions for devil PD-1. Dashes indicate gaps that have been introduced for best-fit multiple-sequence alignments. The putative extracellular domains, transmembrane domains, intracellular domains, immunoreceptor tyrosine-based inhibitory motif, and immunoreceptor tyrosine-based switch motif (ITSM) are denoted by lines above the sequence. The devil PD-1 ITSM shares 100% identity with all species except mouse, opossum, and platypus.

**Figure 2:**
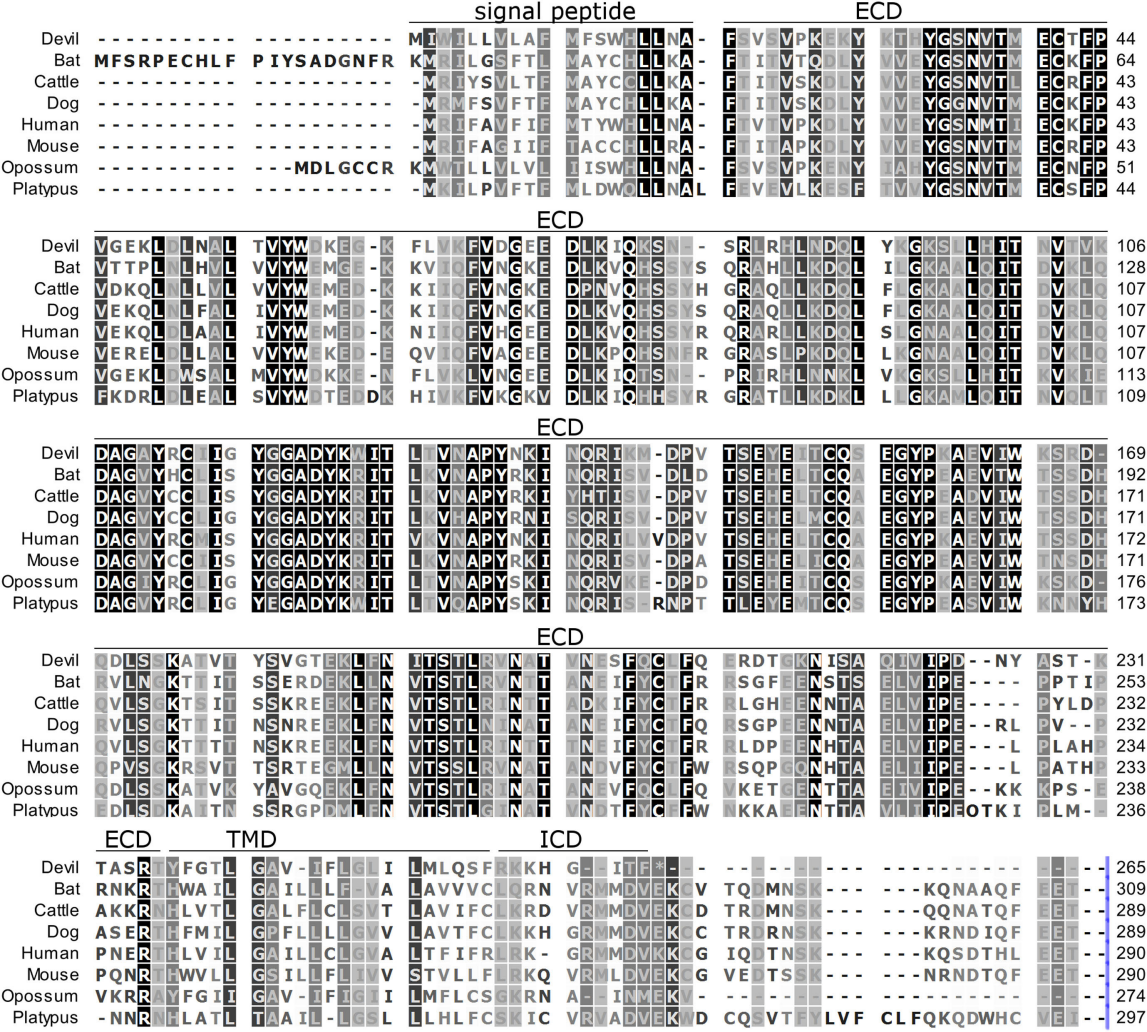
**Alignment of PD-L1 amino acid sequence in eight mammalian species**. The black-to-white color gradient indicates the degree of amino acid conservation among species, with black boxes indicating the most highly conserved sequence regions and white boxes indicating the most divergent regions for devil PD-L1. Dashes indicate gaps that have been introduced for best-fit multiple-sequence alignments. The putative extracellular domains, transmembrane domains, intracellular domains, and signal peptides are denoted by lines above the sequence.

**Figure 3:**
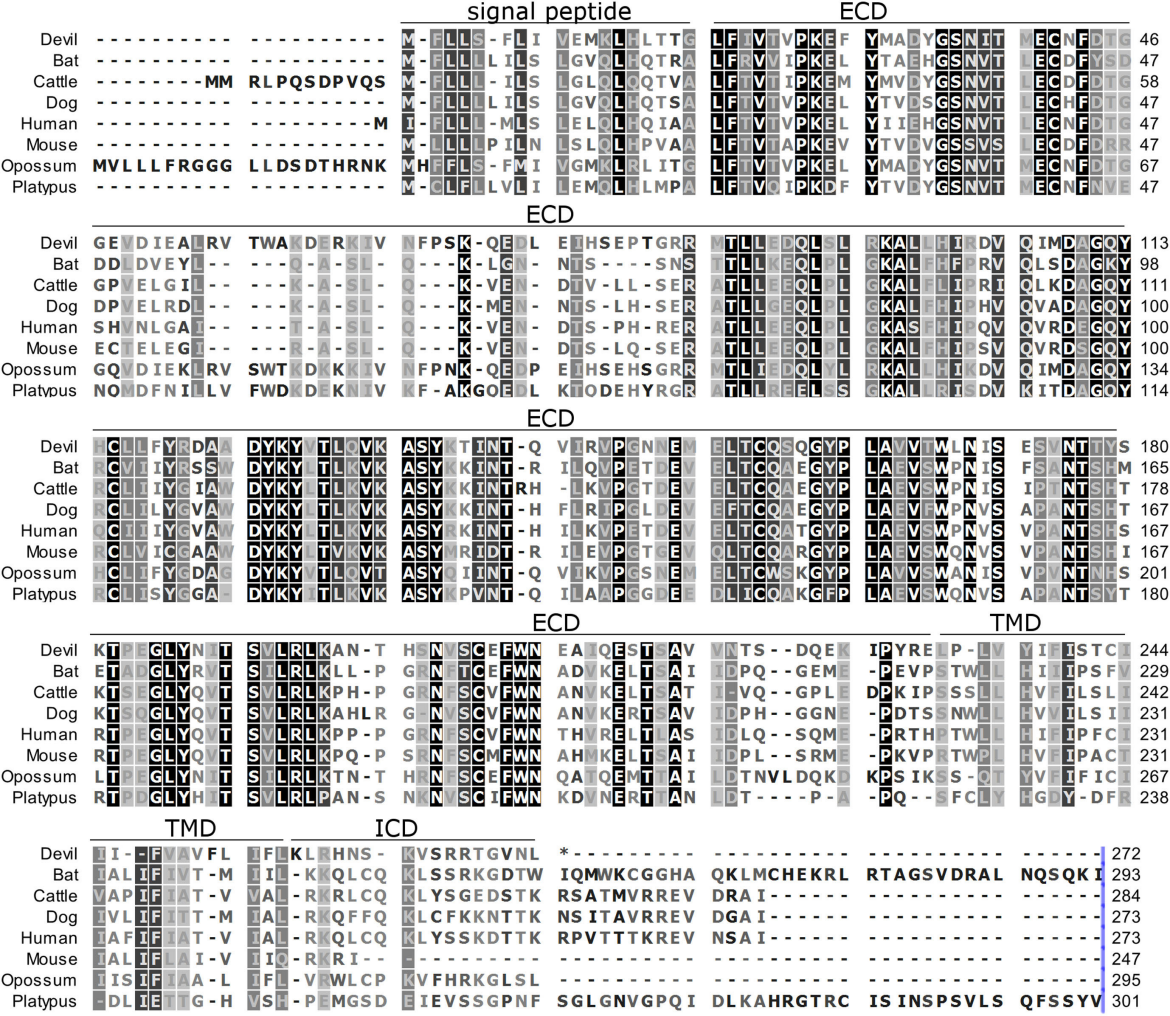
**Alignment of PD-L2 amino acid sequence in eight mammalian species**. The black-to-white color gradient indicates the degree of amino acid conservation among species, with black boxes indicating the most highly conserved sequence regions and white boxes indicating the most divergent regions for devil PD-L2. Dashes indicate gaps that have been introduced for best-fit multiple-sequence alignments. The putative extracellular domains, transmembrane domains, intracellular domains, and signal peptides are denoted by lines above the sequence.

Full-length translated gene sequences for Ensembl transcripts: PDCD1-201 ENSSHAT00000018586 (PD-1), CD274-201 ENSSHAT00000003059 (PD-L1), PDCD1LG2 ENSSHAG00000004176 (PD-L2), and IFNG ENSSHAT00000017850 (IFN-γ) were analyzed using the Phobius web server (28) and TMHMM web server (29) to predict membrane topology and SignalP 4.1 server (30) and Phobius to predict signal peptides. The ExPASy server ProtParam tool (31) was used to predict molecular weight and extinction coefficients, and the Eukaryotic Linear Motif resource (32) was used to predict immunoreceptor tyrosine-based inhibitory motif (ITIM) and immunoreceptor tyrosine-based switch motif (ITSM) sites. Predicted protein structures were confirmed by alignment with protein sequences in several other mammalian species (Figures 1–3) using CLC Sequence Viewer (33) with the “gap open cost” set to 10, the “gap extension cost” set to 1, and the “end gap cost” set to “cheap.” The sequences that we used to make surface-expressed proteins have been deposited in the GenBank database with the following accession numbers: PD-1 (KY075915), PD-L1 (KY075916), and PD-L2 (KY075917).

### PCR on cDNA from PBMCs and Tumor Cells

Primers for PD-1, PD-L1, and PD-L2 were developed for inserting either full-length or extracellular domains (ECDs) into expression vectors (see Table S1 in Supplementary Material for a full list of primers). Total RNA was extracted from DFT1 cell line C5065 with or without 24 h stimulation with supernatant containing devil IFN-γ from CHO cells stably transfected with the pAF23 vector (devil IFN-γ). Devil PBMCs were stimulated overnight with ConA (5 μg/ml) prior to mRNA extraction. mRNA was reverse transcribed into cDNA using ProtoScript II First Strand cDNA Synthesis Kit (New England Biosciences). Qualitative assessment of PD-L1 expression in devil PBMCs and IFN-γ treated and untreated DFT1 C5065 was performed by amplifying a 280 bp segment of PD-L1 from cDNA. GAPDH was used as a reference gene and B2m was used as a positive control for IFN-γ effects. Primers used were as follows: cadeGAPDH_F1 and cadeGAPDH_R1 for GAPDH, B2Mex1F and B2Mex2R for B2m, and deB7H1_318_F and deB7H1_597_R for PD-L1. Reactions were performed following the manufacturer’s recommendations for Kapa HiFi HotStart ReadyMix (Kapa Biosystems # KK2602), and cycling conditions consisted of an initial denaturing step of 95°C for 3 min, followed by 30 cycles of 98°C for 20 s, 60°C for 20 s, and 72°C for 20 s, and a final extension step at 72°C for 5 min. Reaction products were then run on a 1% agarose gel at 100 V.

### Plasmid Construction

pHULK piggyBac mammalian expression vectors (DNA2.0 pJ507-02) were digested with *Bsa*I for 1 h at ambient temperature and gel purified to create linear vectors for cloning. In order to expedite subsequent cloning and development of recombinant fusion proteins, we used overlap PCR extension and T4 ligase cloning techniques to create a modified pAF07 vector that contained bicistronic green fluorescent protein (GFP) and IRES-DsRed, which included additional restriction sites (*Xba*I, *Bam*HI, *Xho*I, *Nhe*I, and *Sal*I). All expression vectors included the Kozak consensus sequence GCCGCCACC upstream of the start codon to ensure efficient initiation of the translation process (34).

In order to test our recombinant protein production approach, we first developed a vector that coded for devil IFN-γ (pAF23). Following confirmation that our recombinant devil IFN-γ was capable of upregulating B2m on of DFT1 cells *in vitro* [in Ref. (11)], we created the pAF27 vector that fused the devil IFN-γ signal peptide, an *Xba*I restriction site, GFP, an a *Sma*I restriction site, a (G_4_S)_2_ linker, a 6xHis-tag, and a *Sal*I restriction site. All subsequent 6xHis-tagged fusion proteins (PD-1-His, PD-L1-His, and IFN-γ-His) were made by digesting pAF27 with *Xba*I + *Sma*I to remove the GFP coding region, and using Gibson Assembly (NEB # E5510S) following the manufacturer’s instructions to insert IFN-γ or the ECDs of PD-1 or PD-L1. Plasmids for full-length molecules including the transmembrane domains and intracellular domains (ICDs) (e.g., PD-L1 full-length) were cloned into *Xba*I + *Sal*I digested pAF07 (see Table S1 in Supplementary Material for primers). Additionally, we generated PD-1-ECD fused to human IgG1-Fc (PD-1-Fc) by digesting a pFUSE vector (Invivogen # pFUSE-hIgG1-Fc2) with *Nco*I and recombinant shrimp alkaline phosphatase (NEB # M0371S) and then using Gibson Assembly to ligate PD-1-ECD into the vector. All assembled plasmids were transformed into NEB 5-alpha competent *Escherichia coli* (NEB # C2987) following the manufacturer’s recommendation. Plasmid DNA was then sequenced by Australian Genome Research Facility (AGRF) and aligned using SnapGene^®^ software (GSL Biotech).

### Transfection and Generation of Recombinant Cell Lines

Stably transfected CHO cell lines expressing full-length surface proteins (PD-1, PD-L1, and PD-L2) and secreted proteins (IFN-γ, PD-1-His, PD-1-Fc, and PD-L1-His) were developed using a protocol modified from Matasci et al. (35) and Hsu and Uludag (36). Briefly, adherent CHO cells were cultured overnight in 6-, 12-, or 24-well plates until the adherent cells reached 40–80% confluency. Plasmid DNA for transfection was diluted in a microfuge tube to 1 μg per 1 × 10^6^ cells to be transfected in 150 mM NaCl or serum-free RPMI and incubated for 2 min. Twenty-five kilodalton linear polyethylenimine (Polysciences # 23966-2) was added to the DNA at a 3:1 ratio, and the solution was vortexed and incubated for 15–30 min at ambient temperature before the solution was added to the adherent cells. The cells were then incubated for 2–4 h before the media were removed and replaced with cRF10. The following day, the transfected cells were subjected to selection with hygromycin (1 mg/ml) for at least 7 days, and then maintained with 0.2 mg/ml of hygromycin.

### Purification of Recombinant Proteins

After reaching 60–80% confluence in T175 flasks in cRF5 medium, the transfected CHO cells secreting recombinant fusion proteins were transferred directly to 50 ml CHO Ex-Cell media (Sigma # 14361C-1000ML) and cultured in magnetically stirred propeller flasks at 75 rpm. The density of cells was then maintained between 5 × 10^5^ and 5 × 10^6^ cells/ml by adding fresh media. Supernatant was collected and centrifuged at 3,200 rcf for 10 min at 4°C to remove cells and debris. The supernatant containing His-tagged proteins was then diluted 1:1 with 20 mM sodium phosphate buffer to a pH of 7.4. The diluted supernatant was then loaded onto HisTrap Excel columns (GE Health Care # 17-3712-05), and the proteins were eluted using 20 mM sodium phosphate buffer with 0.5 M imidazole. Fusion proteins were then dialyzed in PBS using 6–8 kDa dialysis cassettes (Sigma # PURX60005) and then concentrated by evaporation in the dialysis cassette, aliquoted, and frozen until further use. The molecular weight and extinction coefficients for each protein were calculated using the ExPASy Bioinformatics Resource Portal tools (37). Protein concentration was determined using the appropriate molecular weight (kDa) and extinction coefficient (ϵ/1,000) settings on a Nanodrop spectrophotometer. PD-1-Fc was cultured in cRF5, and the supernatant was used directly for *in vitro* FACS analysis.

### Development of Antibodies

Immunization of BALB/c mice for antibody production was approved by the University of South Australia Animal Ethics Committee (# 139/13) and the University of Tasmania Animal Ethics Committee (# A0014680) and was performed in accordance with relevant guidelines and regulations. Mice were immunized subcutaneously on each flank and the back of the neck with a total of 100 μg of fusion proteins (i.e., devil PD-L1-His) in a SqualVax (Oz Biosciences # SQ0010) emulsion on days 0 and 14. At least 14 days after the day 14 booster, the mice were intravenously administered 100 μg of fusion proteins. Four or five days following the intravenous booster, the mice were humanely killed and the spleen and lymph nodes (LN) were harvested. Hybridomas were produced using SP2/0 mouse myeloma cells as the fusion partner and following a standard polyethylene glycol hybridoma fusion protocol (38, 39). Briefly, following hybridoma fusion, the cells were cultured for 1 day in complete Iscove’s Modified Dulbecco’s Medium (IMDM) (1% non-essential amino acids, 100 U/ml penicillin–streptomycin, 2 mM l-glutamine, 50 μM β-ME, and 10 mM HEPES) with 20% FBS, and hybridoma fusion and cloning supplement (Roche # 11363735001). One day postfusion, HAT media supplement (Sigma # H0262-10VL) was added to the culture.

Hybridomas were initially screened for antibody binding to fusion proteins *via* ELISA, and results were confirmed *via* flow cytometry using a FACS Canto II (BD Biosciences) using stably transfected CHO cells (e.g., CHO.PD-L1). Hybridomas were then be subcloned by limiting dilution into 96-well plates complete IMDM with 20% FBS and hybridoma fusion and cloning supplement. Monoclonal cell lines were then screened by ELISA to select lines that produced the highest levels of mAbs. Anti-PD-1 and anti-PD-L1 mAbs were tested for their ability to block receptor:coreceptor interactions (i.e., PD-1:PD-L1) using stably transfected cell lines (CHO.PD-1, CHO.PD-L1, and CHO.PD-L2) and PD-1 fusion proteins.

### Assessment of PD-L1 on Cell Lines

DFT1.C5065, DFT1.1426, DFT1.4096, and DFT2.JV cells were cultured for 3 days with or without 5 ng/ml IFN-γ and then cultured for four additional days without IFN-γ. PD-L1 and PD-1 expression were quantified on days 1, 2, 3, and 7 by flow cytometry. Briefly, DFT cells were blocked with 5% normal goat serum in FACS buffer (PBS with 0.5% BSA, 0.05% NaN_3_) for 15 min on ice in u-bottom 96-well plates, and then incubated with 1 μg/sample of anti-PD-L1 clone 1F8 or anti-PD-1 clone 3G8 for 15 min on ice. The plates were then topped up with 150 μl with FACS buffer and centrifuged at 500 g for 3 min at 4°C. Samples were then incubated with 0.125 μg/sample of goat anti-mouse IgG-phycoerythrin (Abcam # AB98742) for 15 min. The plates were then topped up with 150 μl with FACS buffer and centrifuged at 500 g for 3 min at 4°C and then resuspended in FACS buffer prior to performing flow cytometric analysis using a FACS Canto II (BD Biosciences).

### Immunohistochemistry

Immunohistochemistry staining methods for the tissue sections used here have been previously reported in Kreiss (6), Tovar et al. (40) and Brown (41). Briefly, 3-μm slides were heated for 10 min at 60°C prior to dewaxing in xylene for 5 min (2×) and then rehydrating in 100, 95, and 70% ethanol for 3 min each. Antigen retrieval was carried out by placing the slides in 0.1 M citric buffer in a sealed container and then heating in a pressure cooker for 6 min. The slides were then allowed to cool and rinsed with water before being immersed in 10% H_2_O_2_ for 10 min. The slides were then washed with PBS and non-specific binding was blocked using Dako protein block. Cell culture supernatant (1–5% IMDM) from α-PD-L1 hybridoma clone 1F8 and α-PD-1 hybridoma clone 3G8 were used neat. The slides were then washed with PBS (2×) prior to adding Dako Envision + System HRP-labeled polymers and incubating for 30 min. The slides were again washed in PBS (2×). The Dako liquid DAB + substrate chromogen system (Dako # K3468) was used according to the manufacturer’s instructions. Finally, slides were counterstained with Mayer Hematoxylin for 30 s before washing in running water for 4–5 min, washing for 4–5 min in running water, and submerging in 95% ethanol for 2 min, 100% ethanol for 2 min, and xylene for 2 min (2×). Slides were then coverslipped and stored in the dark until examination.

The cases of DFT2 were previously diagnosed in Pye et al. (3) by histopathology, cytogenetic analysis and detection of specific alleles at microsatellite and structural variant foci different to DFT1 and host DNA, and by PCR. DFT1 was diagnosed based on histopathological and immunohistochemical characteristics (40, 42).

## Results

### Alignment of PD-1, PD-L1, and PD-L2

To determine if PD-1, PD-L1, and PD-L2 have the potential to serve as inhibitory checkpoint molecules in Tasmanian devils as they do in other mammals, we first compared the devil amino acid sequences with sequences from several other mammalian species, including bat (*Myotis davidii*), cattle (*Bos taurus*), dog (*Canis lupus familiaris*), human (*Homo sapiens*), mouse (*Mus musculus*), opossum (*Monodelphis domestica*), and platypus (*Ornithorhynchus anatinus*) (Figures 1–3). Devil PD-1 has 37% sequence identity with human PD-1. The ICD of PD-1 contained putative ITIM and ITSM regions consistent with location and sequence of functional ITIMs and ITSMs in humans and mice (Figure 1); the devil PD-1 ITSM amino acid sequence was an exact match to human PD-1 ITSM, and the ITIM sequence differed by one amino acid between devils and humans. Alignments of PD-L1 showed 55% sequence identity with human PD-L1 (Figure 2). The devil PD-L2 amino acid sequence has 48% sequence identity with human PD-L2 (Figure 3).

### Expression of PD-L1 mRNA in PBMCs and DFT1

**Figure 4:**
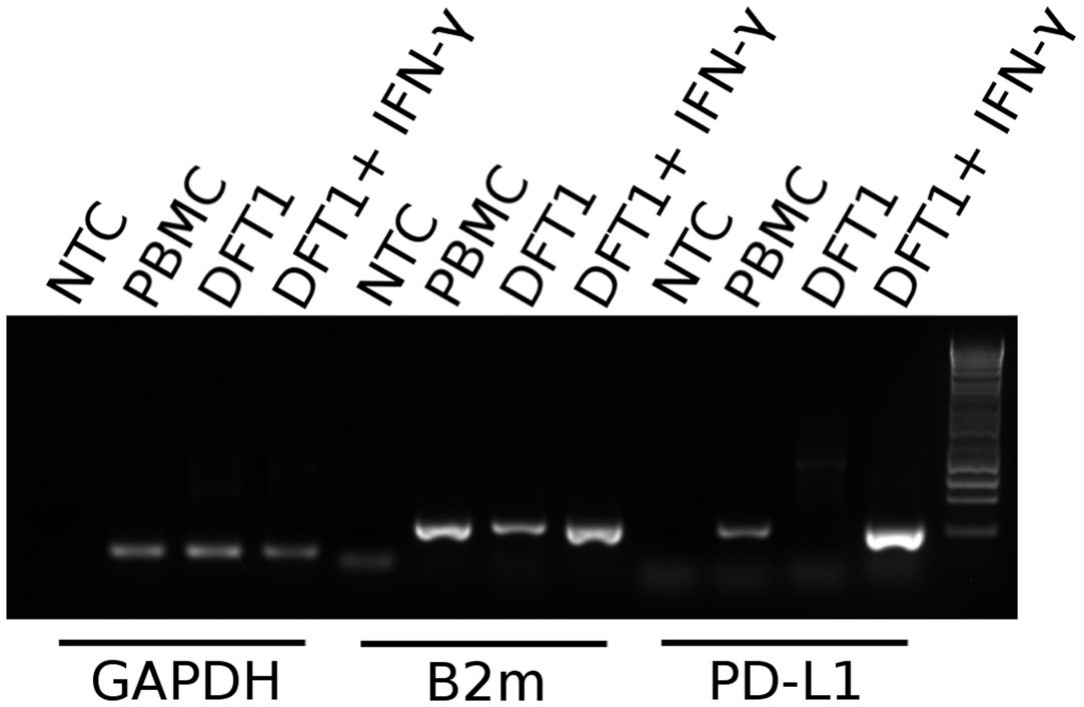
**PD-L1 mRNA expression in PBMCs and devil facial tumor 1 (DFT1) cells**. Expression of PD-L1 in concanavalin A-stimulated PBMCs and DFT1 C5065 cells with and without IFN-γ treatment. The mRNA was reverse transcribed and expression of PD-L1 was tested *via* PCR. GAPDH was used as a reference gene and β2-microglobulin (B2m) was used as a positive control. The unstimulated tumors express only low levels of B2m and PD-L1 transcripts, but both B2m and PD-L1 expression increases following stimulation with IFN-γ. NTC, no template control. The rightmost lane in the gel contains the 1 kb DNA ladder.

Prior to embarking on recombinant protein and mAb production, we sought to establish if, like in better-characterized human and mouse tumor cell lines, devil tumor cells upregulate PD-L1 in response to IFN-γ treatment. PCR of cDNA from DFT1 cell line C5065 demonstrated that PD-L1 is not constitutively expressed in C5065, but that PD-L1 was strongly upregulated following IFN-γ treatment (Figure 4). As expected, B2m was also upregulated by IFN-γ (11).

### PD-1 Binding to PD-L1 and PD-L2 and Blocking Capacity of mAbs

**Figure 5:**
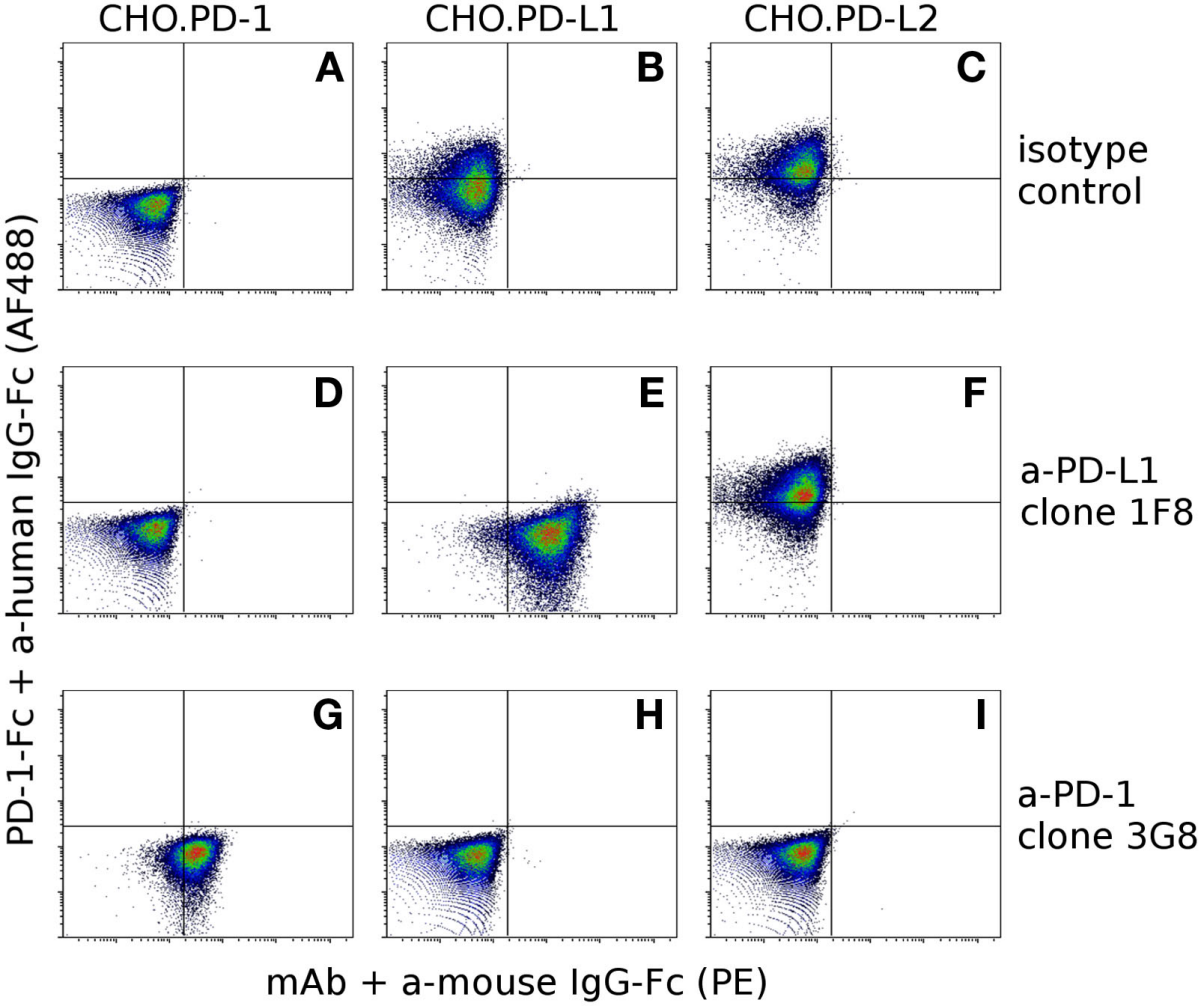
**Confirmation of antibody specificity and blocking capacity**. To assess the specificity and blocking capacity of devil-specific α-PD-L1 monoclonal antibodies (mAbs) CHO.PD-1 **(A,D,G)**, CHO.PD-L1 **(B,E,H)**, and CHO.PD-L2 **(C,F,I)** transfected cells were preincubated with α-PD-L1 mAb clone 1F8 or isotype control antibodies prior to incubation with recombinant PD-1 fused to human IgG1-Fc (PD-1-Fc). Cells were then incubated with secondary anti-human IgG-Fc AlexaFluor 488 and anti-mouse IgG-phycoerythrin to detect PD-1-Fc binding and α-PD-L1 binding. Cells in the upper left quadrant indicate PD-1-Fc binding to the cell line and cells in the lower right indicate binding of mAbs to the cell line. Graphs **(A–F)** show that α-PD-L1 clone 1F8 binds to PD-L1, but not to PD-1 or PD-L2 and that clone 1F8 blocks binding of PD-1-Fc to PD-L1 but not to PD-L2. In order to asses blocking capacity of α-PD-1 mAbs, PD-1-Fc was preincubated with α-PD-1 mAb 3G8 prior to incubation with CHO.PD-1 **(G)**, CHO.PD-L1 **(H)**, or CHO-PD-L2 **(I)**. Graphs **(G–I)** show that α-PD-1 clone 3G8 is specific for PD-1 and blocks binding of PD-1-Fc to both PD-L1 and PD-L2.

Our hybridoma development process yielded 10 α-PD-1 mAbs and 9 α-PD-L1 mAbs. Six of the nine α-PD-L1 mAbs could block binding of PD-1-Fc fusion proteins to CHO.PD-L1, but none of the α-PD-L1 mAbs blocked binding of PD-1 fusion proteins to CHO.PD-L2 (Figure 5). Five of the anti-PD-1 mAbs block binding of PD-1-Fc to both PD-L1 and PD-L2 (Figure 5) and two of the clones appear to partially block PD-L1 but not PD-L2 (Figure S1 in Supplementary Material). See Table S2 in Supplementary Material for complete details for the α-PD-1 and α-PD-L1 panel of mAbs.

### *In Vitro* Expression of PD-L1 on DFT Cells

**Figure 6:**
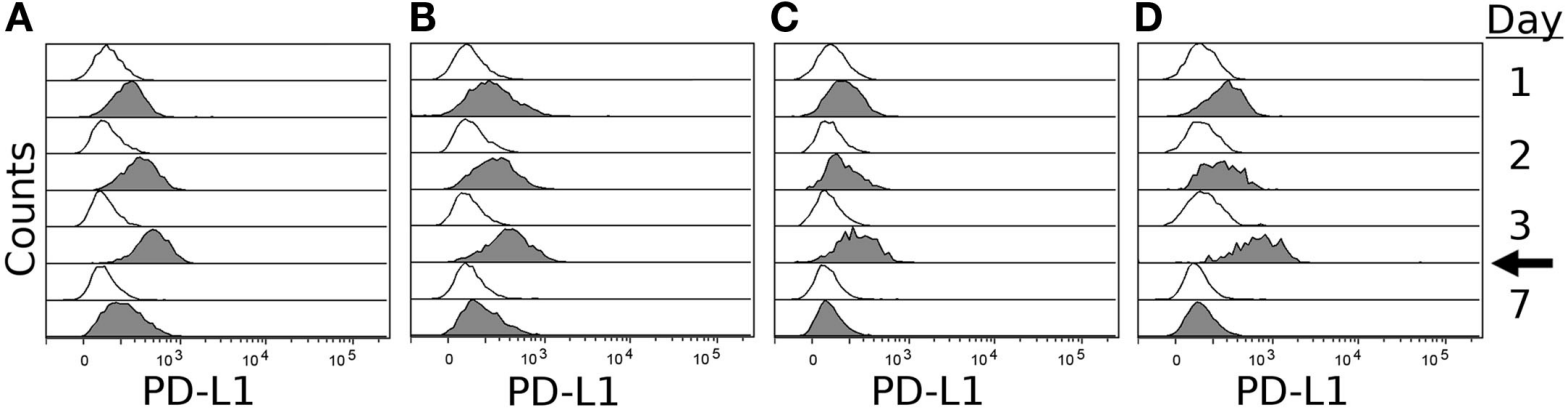
**IFN-γ induced expression of PD-L1 on devil facial tumor 1 (DFT1) and devil facial tumor 2 (DFT2)**. DFT1 cell lines 1426 **(A)**, 4096 **(B)**, and C5065 **(C)** and DFT2 cell line JV **(D)** were stimulated with 5 ng/ml of IFN-γ for 3 days, and PD-L1 surface expression was assessed on days 1, 2, 3, and 7. PD-L1 surface expressions were highest at day 3 post-IFN-γ exposure, and PD-L1 returned to near baseline levels by day 7 (4 days after removal of IFN-γ). The arrow indicates when IFN-γ was removed from the culture. Gray histograms represent cells treated with IFN-γ and open (white) histograms represent untreated cells.

To confirm expression of PD-L1 on the surface of DFT cell lines, we stimulated four DFT cell lines (DFT1.C5065, DFT1.1426, DFT1.4096, and DFT2.JV) with IFN-γ for 3 days and then used flow cytometry to quantify PD-L1 expression on days 0, 1, 2, 3, and 7 using α-PD-L1 (1F8). All cell lines expressed little or no PD-L1 on day 0, but upregulated PD-L1 following IFN-γ treatment (Figure 6). PD-L1 expression increased until day 3 when IFN-γ was removed from the cultures, but was still detectable on day 7, which is 4 days after IFN-γ was removed from the cultures. PD-1 was not expressed on either DFT1 or DFT2 with or without IFN-γ.

### Expression of PD-1 and PD-L1 in Devil Tissues

**Figure 7:**
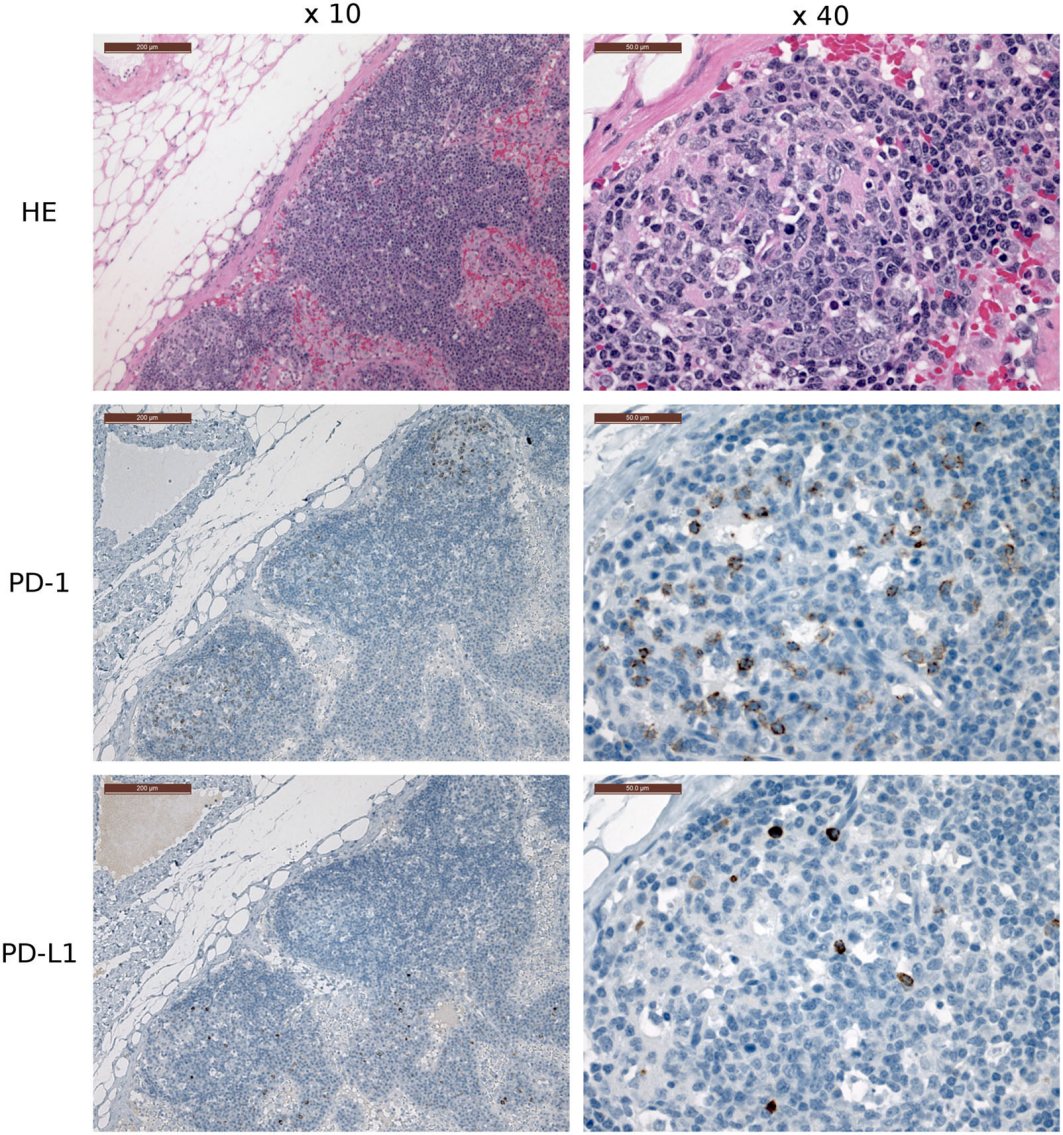
**Immunohistochemistry (IHC) of devil lymph nodes (LN)**. IHC of LN from a devil facial tumor 1 (DFT1) + devil (TD 374). The scale bar in the upper left of each ×10 image is 200 μm and the ×40 images are 50 μm. No tumor metastases were observed in the LN shown in this figure. PD-1 is expressed on 0–10% of lymphocytes in the cortical germinal centers but less than 1% of non-germinal center lymphocytes. PD-L1 expression is distributed throughout the cortex of the LN, but was detected on less than 1% of lymphocytes (at ×40 objective).

**Figure 8:**
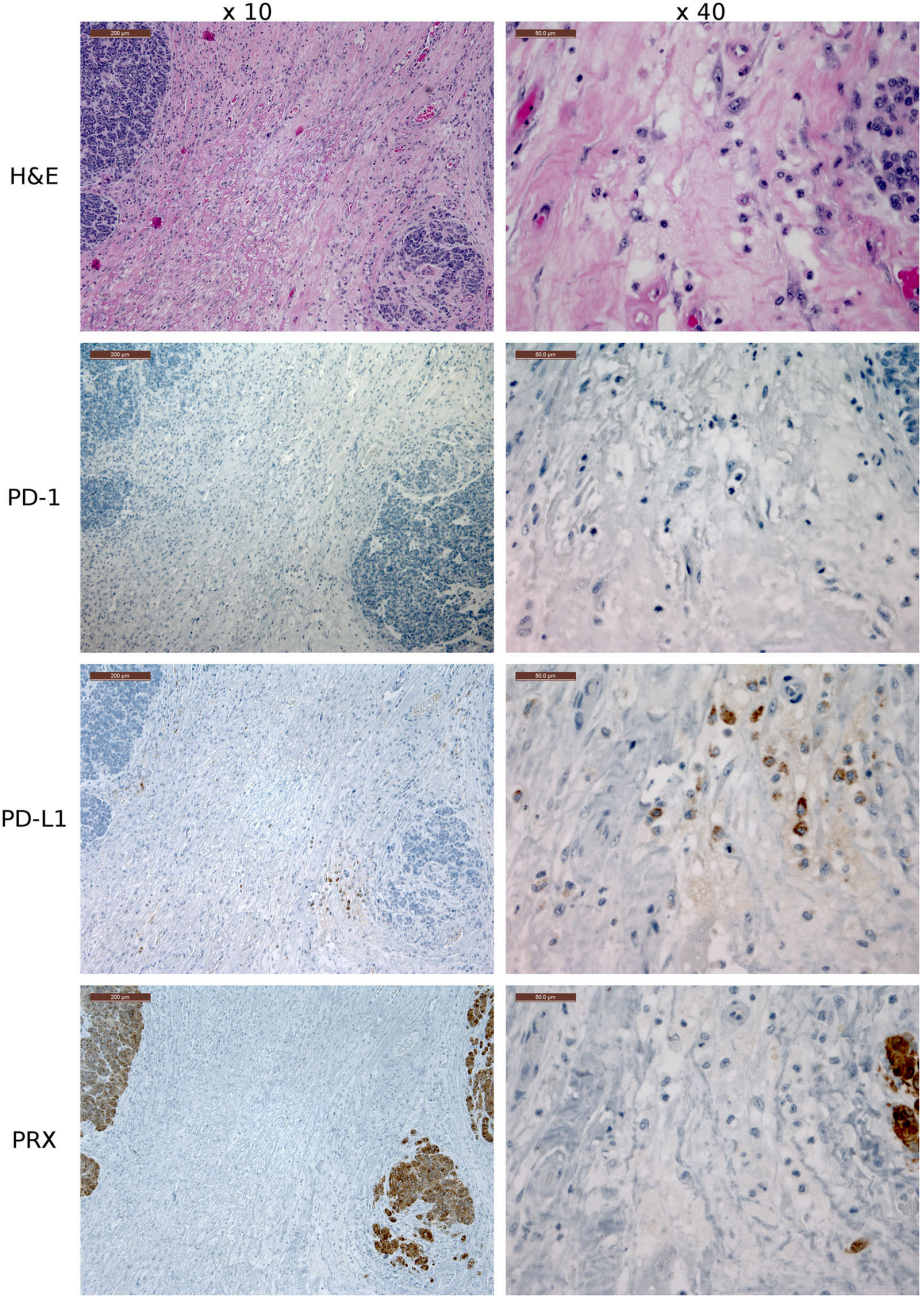
**Immunohistochemistry (IHC) devil facial tumor 1 (DFT1) tissue**. IHC images of DFT1 tissue sections from TD 512 using the ×10 and ×40 objectives. The scale bar in the upper left of each ×10 image is 200 μm and the ×40 images are 50 μm. The bottom row is stained with α-Periaxin, which is used to identify DFT1 cells. PD-L1 was occasionally detected on tumor cells but was more commonly detected in low numbers of lymphocytes, plasma cells, and macrophages within the fibrovascular stroma supporting multiple lobules within the tumor.

**Figure 9:**
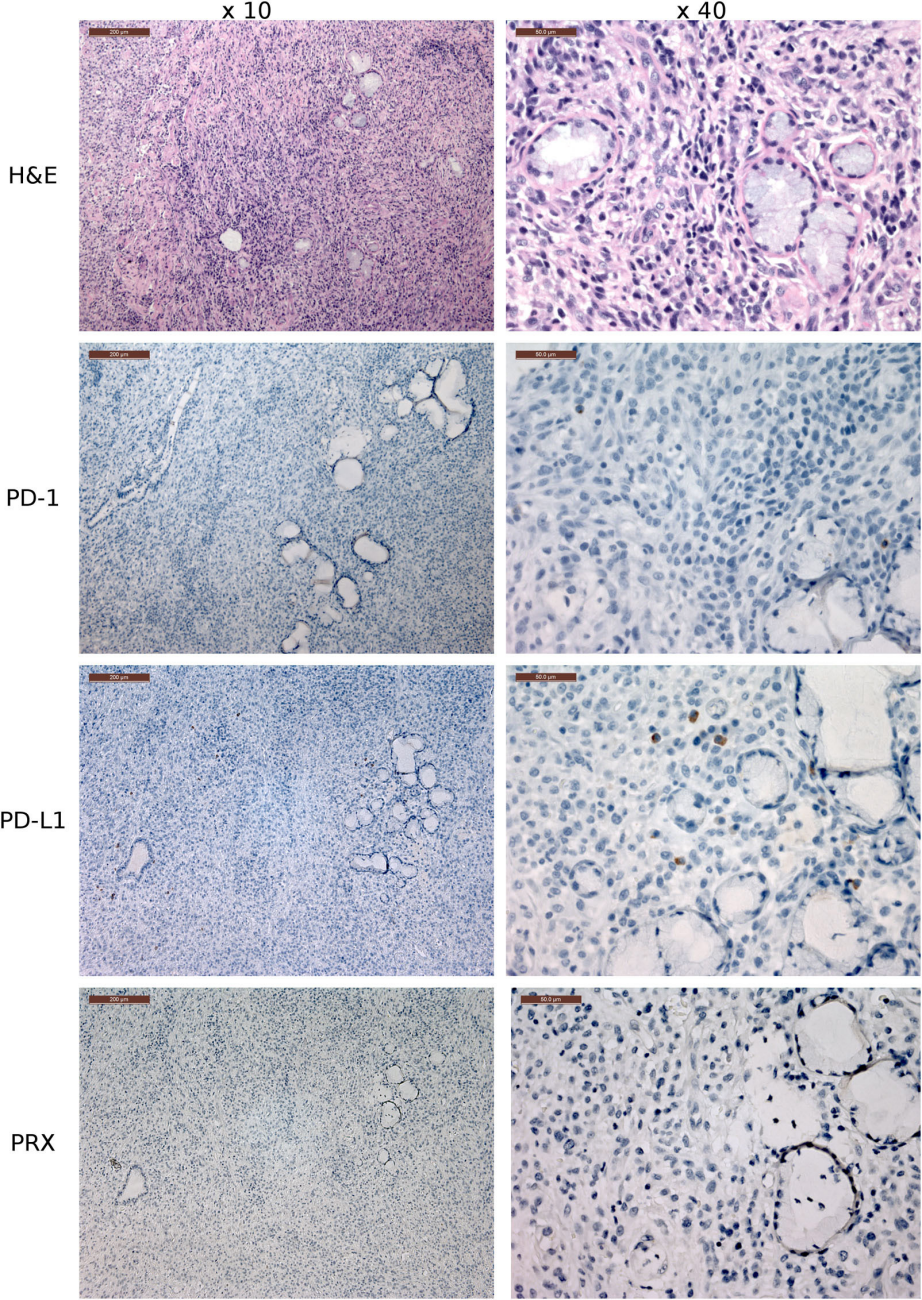
**Immunohistochemistry (IHC) of the fibrovascular interstitium of devil facial tumor 2 (DFT2) tissue**. IHC images of tissue sections from a DFT2 tumor mass removed from TD 500. The scale bar in the upper left of each ×10 image is 200 μm and the ×40 images are 50 μm. Low numbers of plasma cell-like cells with positive cytoplasmic staining for PD-L1 were detected within the fibrovascular interstitium and not infiltrating within tumor packets using the ×10 and ×40 objectives. PD-1^+^ cells were rare throughout the tissue, with only two PD-1^+^ cell detected in the image above. Periaxin (PRX) is shown here for comparative purposes only, as the DFT2 cells do not express PRX.

**Figure 10:**
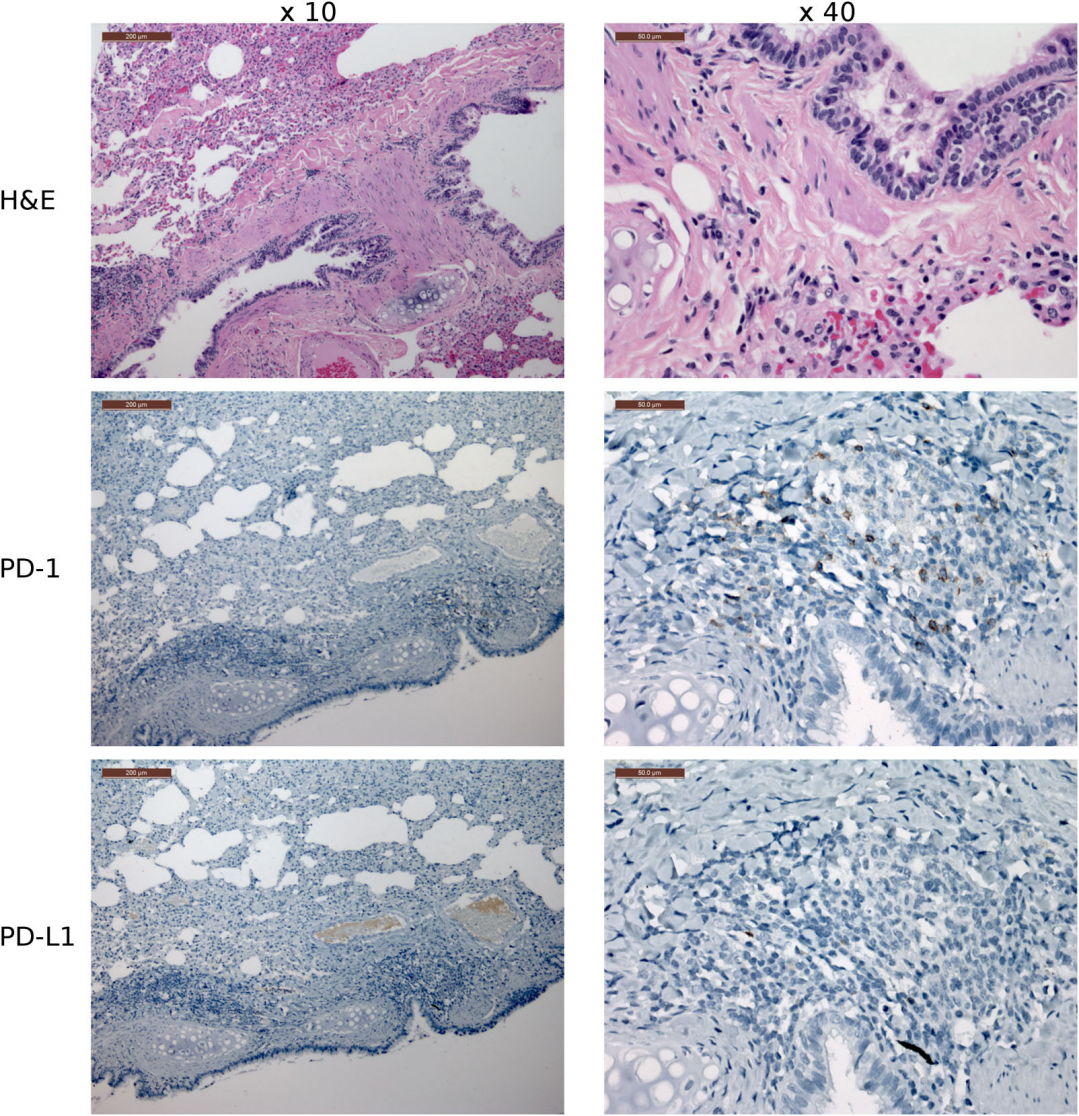
**Immunohistochemistry (IHC) lung tissue with devil facial tumor 2 (DFT2) metastases**. IHC images of lung tissue from TD467. DFT2 metastases are not visible in the images shown here. The scale bar in the upper left of each ×10 image is 200 μm and the ×40 images are 50 μm. The lungs contained low numbers of lymphocytes with positive cytoplasmic staining for PD-1, but the lymphocytes are predominantly peribronchiolar and not near tumor metastases. PD-L1 was not detected on tumor cells and was rare on non-tumor cells.

Several of the α-PD-1 and α-PD-L1 mAbs were capable of staining of FFPE tissue sections. Clones 3G8 and 1F8 were used for IHC because these clones also block PD-1:PD-L1 binding in flow cytometric assays and are thus the initial candidates for immunotherapy treatment of devil tumors. Low numbers of lymphocytes (1–2% of lymphocytes per high-powered field, that is at ×40 objective) in the outer cortex of LN generally demonstrated positive cytoplasmic staining for PD-L1. A few germinal centers contained 5–10% PD-L1^+^ lymphocytes per high-powered field (at ×40 objective) (Figure 7). PD-1 was rarely detected in lymph node structures other than germinal centers. We screened tumor-containing tissue sections from 18 devils *via* IHC that were either naturally infected (*n* = 12) or experimentally inoculated (*n* = 5) with tumors as part of a different study (1) and found that PD-L1 was generally not expressed in DFT1 (Figure 8) or DFT2 (Figure 9) cells except for a few sporadic cells. Twenty out of 33 tumor-containing tissue sections contained one or more PD-L1^+^ cells, with the majority of sections containing less than 20 PD-L1^+^ cells. Low numbers of PD-L1^+^ lymphocytes, plasma cells, and macrophages were detected in the fibrovascular interstitium, renal cortical interstitium, cortex and medulla of LN, and peribronchiolar connective tissue near or within tumors in the facial region, kidneys, LN, and lungs, respectively. Interestingly, groups of PD-1^+^ cells were detected in lung tissue of a devil with DFT2 metastases in the lung (Figure 10). See Table S3 in Supplementary Material for a summary of IHC findings.

## Discussion

The transmissible DFTD represents an immunological enigma due to the fact that these transmissible tumor allografts are not recognized and rejected as foreign. This is despite genetic differences between the host and tumor and the high mutational load that has accumulated in the DFT1 in 20 years of host-to-host transmission (9). In humans and mice, expression of PD-L1 on tumor cells represents a powerful immune evasion pathway commonly employed by tumor cells, but the role of PD-1 and PD-L1 had not been previously investigated in devils and transmissible tumors. Here, we provide the first evidence in a non-placental mammalian species that the inhibitory cell surface signaling molecule PD-1 binds to PD-L1 and PD-L2 and that PD-L1 is strongly upregulated on DFT1 and DFT2 cells in response to IFN-γ emphasizing the conserved nature of this signaling module across a range of evolutionarily disparate species.

Analysis of the PD-1 protein sequence revealed putative ITIM and ITSM domains, which are critical for the inhibitory effects of PD-1 observed in other species. Their conservation suggests that PD-1 may serve as a negative regulator to immune cells in devils as it does in humans and mice. The devil PD-1 ITSM sequence was a perfect match to human, dog, bat, and cattle PD-1 ITSM, and the ITIM sequence varied by only a single amino acid, suggesting that strong selective pressure has maintained these motifs across the 160 million years since marsupials and placental mammals diverged (43). Further research is needed to confirm the inhibitory function of devil ITSMs and ITIMs.

In humans and mice, PD-L1 and PD-L2 have different expression patterns and functional effects upon PD-1 binding (44). The panel of dual blocking, single blocking, and non-blocking mAbs developed here will allow us to explore the functional roles of PD-1, PD-L1, and PD-L2 in devils and may help to shed light on immune evasion by cancers and the role of PD-1 and PD-L1 in allograft rejection that is relevant to humans and other species. For instance, the devil immune system rejects skin allografts but not skin autografts or tumor allografts (8), thus suggesting that the tumor cells evade an allo-antigen immune response. As DFTs are primarily transmitted through biting, it is likely that tissue damage is associated with at least some of the transmission events. This could result in an “immune-active” environment and upregulation of PD-L1 (45). Transplantation of DFT1 and DFT2 to hosts with varying degrees of genetic relatedness could shed light on the importance of PD-L1 in tissue damage and inflammation during the early stages of allograft tolerance or rejection and tumor transmission and development. Furthermore, the study of allograft tumors in humans is only possible when tumor cells are unwittingly transplanted into a new host along with the target transplant tissue (46). The clonal transmissibility of the DFTs allows for natural serial transfer experiments using cells that have never been cultured *in vitro*, which could have lasting effects on tumor phenotype, and also facilitates assessment of the roles of PD-1, PD-L1, and PD-L2 in tumor transmission, development, and metastases. Finally, recent evidence suggests that some devils can mount an immune response to DFT tumors (47), and the new reagents developed here could clarify the role of PD-L1 in responders versus non-responders.

In humans, PD-1 is transiently upregulated following activation of naïve T cells, and its expression decreases as the antigen is cleared, but PD-1 expression can be sustained during chronic infections [reviewed in Ref. (22)]. DFTD is essentially a chronic infection that usually kills infected devils within 6–12 months (48), during which time tumor antigens would be abundantly expressed. In the LN that we examined *via* IHC, PD-1 was generally expressed on 1–2% of lymphocytes in lymph node germinal center cells, which is consistent with patterns that have been reported in humans (49–53) and rhesus macaques (*Macaca mulatta*) (54, 55). Unfortunately, because devils are an endangered species, we only had LN from unhealthy devils and thus were unable to compare PD-1 and PD-L1 expression patterns between healthy and diseased devils at this time.

Our *in vitro* results demonstrate that IFN-γ transiently upregulates PD-L1 on both DFT1 and DFT2 cell lines. Our IHC results are in agreement with the *in vitro* results showing that both DFT1 and DFT2 cells do not normally express PD-L1 or express very low levels of PD-L1. This is not surprising given that tumor-infiltrating lymphocytes (TILs) are rarely observed in DFT1 or DFT2 tumors, so local production of IFN-γ by T cells is likely absent in most cases (11, 56, 57). Additionally, upregulation of PD-L1 in response to IFN-γ lasts only a few days. However, as immunity to the tumor cells may be compartmentalized in the draining LN and tumor microenvironment, tumor metastases to new locations might induce an inflammatory response that could result in transient upregulation of PD-L1. We did observe PD-L1^+^ non-tumor cells in the tumor microenvironment of several metastatic tumors and scattered PD-L1^+^ tumor cells.

In humans, the combination of α-CTLA4 and α-PD-1 has generally proven much more effective that either treatment alone (58–60). α-CTLA4 treatment is particularly useful in PD-L1-negative tumors with a high number of neoantigens in which the α-CTLA4 treatment is hypothesized to enhance T cell activation and increase the probability of TILs and stimulate IFN-γ production, at which point combination treatment with α-PD-1 or α-PD-L1 becomes critical for amplifying and maintaining the antitumor response (61, 62). Additional research is needed to determine if CTLA4 serves as an inhibitory checkpoint molecule in marsupials and also if the CD80 binds to both CTLA4 and PD-L1 as it does in humans and mice (63).

In conclusion, this study is the first to show that receptor–ligand interactions between the potent cosignalling molecules PD-1, PD-L1, and PD-L2 are conserved in marsupials. Analysis of PD-1 protein sequence revealed putative ITIM and ITSM domains, which are critical for the inhibitory effects of PD-1 observed in other species. We report that PD-L1 expression is rare on both DFT1 and DFT2 cells, but that PD-L1 expression is upregulated by treatment with IFN-γ. Mapping the complex interactions and expression patterns of inhibitory checkpoint molecules will be critical for developing a DFTD vaccine and understanding how transmissible tumors evade host immune responses. Understanding the immune evasion mechanisms employed by these transmissible tumors could help to shed light not only on cancer immunology but also for transplant tolerance in many species including humans. Finally, the current iteration of the DFT disease vaccine uses DFT cells that have been stimulated with IFN-γ to upregulate MHC I prior to killing the cells and injecting with adjuvant. This treatment has proven effective at stimulating both humoral and cytotoxic responses against tumor cells and has provided short-term protection against tumor challenge (7). The results reported here demonstrate that the PD-L1 is strongly upregulated by IFN-γ and thus blocking PD-L1 prior to administration of the vaccine, or administering α-PD-1 or α-PD-L1 in conjunction with the vaccine, could augment antitumor responses by the devil immune system. Additionally, as both prophylactic and therapeutic DFTD vaccines are high priority, the results presented here represent an important first step toward understanding the complex network of molecular signaling that is necessary for the foreign tissue grafts to avoid being killed by host immune defenses.

## Author Contributions

AF performed all lab experiments, developed proteins and antibodies, analyzed data, prepared the figures, and wrote the manuscript. AL, LC, JM, and AP provided intellectual input and revised the manuscript. GW provided intellectual input, laboratory support, and contributed to writing and revision of the manuscript. JH provided intellectual input, laboratory support, and contributed to the revision of the manuscript. GK reviewed the histopathology slides and contributed to writing and revising the manuscript.

## Conflict of Interest Statement

The authors declare that the research was conducted in the absence of any commercial or financial relationships that could be construed as a potential conflict of interest.

## Abbreviations

DFTD, devil facial tumor disease; DFT1, devil facial tumor 1; DFT2, devil facial tumor 2; cRF10, complete RPMI supplemented with 10% FBS; cRF5, complete RPMI supplemented with 5% FBS; mAbs, monoclonal antibodies; ECDs, extracellular domains; TMDs, transmembrane domains; ICDs, intracellular domains.
