# Supplementary material for: PD-L1 Is Not Constitutively Expressed on Tasmanian Devil Facial Tumor Cells but Is Strongly Upregulated in Response to IFN-γ and Can Be Expressed in the Tumor Microenvironment
Source: Front Immunol. 2016 Dec 9;7:581. doi: 10.3389/fimmu.2016.00581 (PMC5145852; doi:10.3389/fimmu.2016.00581)
Supplement: Supplementary file 1 [file data_sheet_1.pdf]

Supplementary Table 1. Primers used for amplifying Tasmanian devil DNA and plasmid construction.

Name	Sequence	Primer use	Plasmid	Length	Tm
deB7H1_sig-pep_F	ATGATTTGGATTTTGCTTGTGC	PD-L1 5' signal peptide	NA	22	54
deB7H1-short_795_R	TTAAAATGTAATACCATGTTTTTTTCTAAAGC	PD-L1 3' based on cDNA ENSSHAT00000003059	NA	32	54
pAF22_fragment.FOR	agaccacacccaagctgtctagagccgccaccATGATTTGGATTTTGCTTGTGC	Amplify PD-L1 with extensions for plasmid construction	pAF48 (full-length PD-	54	74
pAF48.REV	gaagacgcttttagagaccGTCGACTTAAAATGTAATACCATGTTTTTTTCTAAAGC	Amplify full-length PD-L1 with extensions for cloning	pAF48 (full-length PD-	57	66
pAF38.FOR	GTTCTTCTGGCTGTCTTTCTTTTTCTGTATCTGTTCCCAAAGAGAAATATAAAACAC	Amplify PD-L1-ECD with extensions for 6xHis plasmid construction	pAF38 (secreted	57	66
pAF38.REV	CCTCCACTACCTCCACCCCCAGTTCTTGAGGCTGTCTTAGTTGAT	Amplify PD-L1-ECD with extensions for 6xHis plasmid construction	pAF38 (secreted	45	71
pAF42.FOR	TGTCACGAATTCGATATCGGaCTTTTCTGTATCTGTTCCCAAAGAG	Amplify PD-L1-ECD with extensions for human IgG1-Fc plasmid construction	pAF43 (secreted	46	66
pAF42.REV	GTGAGTTTTGTCAGATCTAACAGTTCTTGAGGCTGTCTTAGTTGAT	Amplify PD-L1-ECD with extensions for human IgG1-Fc plasmid construction	pAF43 (secreted	46	65
pAF36.FOR	agagaccacacccaagctgtgccgccaccATGTTCTTCCTTCTCTCCTTTCTGATTGT	amplify PD-L2 with extensions for plasmid construction	pAF36 (full-length PD-	58	75
pAF36.REV	gaagacgcttttagagaccGTCACAAGTTCACACCAGTTCTGC	amplify full-length PD-L2 with extensions for plasmid construction	pAF36 (full-length PD-	43	69
dePD1.FOR	ATGGTGAAAACCGAATATACCCTTGGCTTTTGGCCAC	amplify PD-1, includes start codon	NA	37	67
dePD1.REV	TCAGAGTGGGCAGGATCCAGGTGGGAG	amplify full-length PD-1 includes stop codon	NA	27	67
pAF24_fragment.FOR	agaccacacccaagctgtctagagccgccaccATGGTGAAAACCGAATATACCCT	amplify PD-1 with extensions for plasmid construction	pAF54 (full-length PD-	55	74
pAF53.REV	gaagacgcttttagagaccGTCGACTCACTTCCGATGGGGAGAGCTG	amplify full-length PD-1 with extensions for plasmid construction	pAF54 (full-length PD-	47	72
pAF39.FOR	GTTCTTCTGGCTGTCTTTCTATGGTGAAAACCGAATATACCCTTGG	Amplify PD-1-ECD with extensions for 6xHis plasmid construction	pAF38 (secreted	46	67
pAF39.REV	CCTCCACTACCTCCACCCCCCTTTTCGGATTGTGCTTGGGTGG	Amplify PD-1-ECD with extensions for 6xHis plasmid construction	pAF38 (secreted	43	74
pAF43.FOR	GTCACGAATTCGATATCGGCaGTGAAAACCGAATATACCCTTGGCTTTTG	Amplify PD-1-ECD with extensions for human IgG1-Fc plasmid construction	pAF43 (secreted	50	69
pAF43.REV	GTGAGTTTTGTCAGATCTAACCTTTTCGGATTGTGCTTGGGTGGTGA	Amplify PD-1-ECD with extensions for human IgG1-Fc plasmid construction	pAF43 (secreted	47	69

deIFNg_seq.FOR	GCAAACTCTTCACAACTACTTTAATGC	IFN-g sequencing and qPCR	NA	27	63
deIFNg_seq.REV	AGCTTTCCTTTGGACTTTGAGG	IFN-g sequencing and qPCR	NA	22	63
pAF29a.FOR	GTTCTTCTGGCTGTCTTTCTCAAGTTAACCTAAGAGAAGACATGC	IFNg 5' insert for pAF29	pAF29	45	66
pAF29a.REV	CCTCCACTACCTCCACCCCCCTGTGTGATTTTTCCTTGGCTTTTGTTC	IFNg 3' insert for pAF29	pAF29	48	72
pAF23_Fragment.FOR	gaccacacccaagctgtctagagccgccaccATGAATTATTCAAGCTACCTCTTAGC	IFNg 5' insert for pAF23	pAF23	57	73
pAF23_Fragment.REV	gaagacgcttttagagaccGTCGACCTACTGTGTGATTTTTCCTTGGC	IFNg 3' insert for pAF23	pAF23	48	70
pAF07_Fragment 1.FOR	cgtgaacacgtctcgggggagagaccacacccaag	Comet GFP with overlaps for pAF07	pAF07	36	70
pAF07_Fragment 1.REV	TCGGATCCgcggtatgtttccaagtccacc	Comet GFP with BamHI + devil IgG hinge region overlap for pAF07	pAF07	30	66
pAF07_Fragment 2.FOR	ataccgcGGATCCGAGGGCCCCAGTTCCACACC	Devil IgG overlap with Comet GFP for pAF07	pAF07	33	72
pAF07_Fragment 2.REV	AGGGGCGATTTAAATTTCCTCGAGTCATGAATTCGAGATTGTCCTCTG	Devil IgG and overlap with IRES for pAF07	pAF07	48	66
pAF07_Fragment 4.FOR	CTTTGAAAAACACGATGATAATATGGCTAGCATGGATAGCACTGAGAACG	DsRed with IRES overlap and NheI site for pAF07	pAF07	50	65
pAF07_Fragment 4.REV	agaacaggaagacgcGTCGACTCACTGGAACAGGTGG	DsRed with SalI site for pAF07	pAF07	37	69
pAF07_Fragment3.FOR	TGACTCGAGGAAATTTAAATCGCCCCTCTCC	IRES with BamHI site for pAF07	pAF07	31	64
pAF07_Fragment3.REV	GCTAGCCATATTATCATCGTGTTTTTCAAAGGAAAACCA	IRES with ATG-NheI site for pAF07	pAF07	39	63
pAF07RE_fragment.FOR	atttcaggtgtcgtgaacacgtctcgggggagagaccacacccaagctg	pAF07 insert for BsaI restriction cloning	pAF07	49	75
pAF07RE_fragment.REV	gatgtgatgagaacaggaagacgcttttagagaccGTCGACTCACTGGAACAGGTGGTG	pAF07 insert for BsaI restriction cloning	pAF07	59	73
pAF27_SigPep_1.FOR	gccgccaccATGAATTATTCAAGCTACCTCTTAGC	IFNg 5' signal peptide with Kozak-ATG	pAF27	35	66
pAF27_SigPep_1.REV	caccttctgtcagggccgtTTCTAGAGAAAGACAGCCAGAAGAACTCAG	IFNg 3'	pAF27	49	71
pAF27_GFP_2.FOR	TCTAGAAacggccctgacagaaggtg	IFNg 5' signal peptide with overlap for comet GFP	pAF27	26	63
pAF27_GFP_2_a.REV	TCCACCCCCGGGgcggtatgtttccaagtccac	Comet GFP 3' and G4S-His extension	pAF27	33	72

pAF27_G4S_His_3.FOR	gaaacataccgcaCCCGGGGAGGTGGAGGTAGTGGAGGTGGAGGTAGTCATCATCACC	Comet GFP 3' and G4S-His extension	pAF27	58	76
pAF27.REV	gaagacgcttttagagaccGTCGACTCAATGGTGATGGTGATGATGACTACCTCCAC	pAF27 3' insert	pAF27	57	72
pAF27.FOR	gaccacacccaagctgtgccgccaccATGAATTATTCAAG	pAF27 5' insert	pAF27	40	70
pAF27_G4S_His_3_a.FOR	acataccgcCCCGGGGGTGGAGGTAGTGGAGGTGGAGGTAGTCATCATCACCATCAC	Comet GFP 3' and G4S-His extension	pAF27	57	77
pAF27_GFP_2_a.REV	TCCACCCCCGGGgcggtatgtttccaagtccac	Comet GFP 3' and G4S-His extension	pAF27	33	72
pFUSE_seq.FOR	GACCGGCGCCTACCTGAGAT	sequencing primers for the human and mouse pFUSE-Ig-Fc plasmids	sequencing	20	62
pFUSE_seq.REV	CATCAATGTATCTTATCATGTCTGGCCAG	sequencing primers for the human and mouse pFUSE-Ig-Fc plasmids	sequencing	29	59
pb_EF1a_seq_f	atcttggttcattctcaagcctcag	Sequencing pHULK piggybac plamids	sequencing	25	59
pb_scaffold_seq_R	aggctaagtaacatctgtggca	Sequencing pHULK piggybac plamids	sequencing	24	58
PCR-IRES-Fw	GAAATTTAAATCGCCCCTCTCCC	IRES with 5' extension to avoid GC repeats	sequencing	20	57
PCR-IRES-Rv	ATTATCATCGTGTTTTTCAA	IRES	sequencing	20	55

Supplementary Table 2. Blocking capacity of PD-1 and PD-L1 monoclonal antibodies

antigen	clone	antibody_ID	target species	host species	antagonist
PDL1	1F8	PDL1_1F8	devil	mouse	yes, blocks PD1-his to PDL1 20150701 and PD1-Fc to PDL1 but not PDL2 20151105
PDL1	2A11	PDL1_2A11	devil	mouse	yes, blocks PD1-his to PDL1 20150701 and PD1-Fc to PDL1 but not PDL2 20151105
PDL1	2E4	PDL1_2E4	devil	mouse	partial block of PD1-His 20150701, does not block PD1-Fc binding to PDL1 or PDL2 20151105.
PDL1	2G2	PDL1_2G2	devil	mouse	no, does not block PDL1 20150701 or PDL2 20151105
PDL1	2H5	PDL1_2H5	devil	mouse	yes, blocks PD1-his to PDL1 20150701 and PD1-Fc to PDL1 but not PDL2 20151105
PDL1	3G4	PDL1_3G4	devil	mouse	no, does not block PDL1 20150701 or PDL1 and PDL2 20151105
PDL1	4A10	PDL1_4A10	devil	mouse	yes, blocks PD1-his to PDL1 20150701 and PD1-Fc to PDL1 but not PDL2 20151105
PDL1	4A12	PDL1_4A12	devil	mouse	yes, blocks PD1-his to PDL1 20150701 and PD1-Fc to PDL1 but not PDL2 20151105
PDL1	5D11	PDL1_5D11	devil	mouse	yes, blocks PD1-his to PDL1 20150701 and PD1-Fc to PDL1 but not PDL2 20151105
PD1	1B6	PD1_1B6	devil	mouse	yes, blocks PD1-his to PDL1 20150701 and PD1-Fc to PDL1 and PDL2 20151105
PD1	1C9	PD1_1C9	devil	mouse	no. Does not block PD1-His to PDL1 20150701 and PD1-Fc to PDL1 and PDL2 20151105
PD1	3C4	PD1_3C4	devil	mouse	partial block of PD-1 His and huIgG-Fc to PDL1 but not PDL2
PD1	3G8	PD1_3G8	devil	mouse	yes, blocks PD1-his to PDL1 20150701 and PD1-Fc to PDL1 and PDL2 20151105
PD1	4E3	PD1_4E3	devil	mouse	yes/no. doesn't block PD-1-His 20150701 to PDL1, blocked PD-1-Fc on 20151105 to PDL1 and PDL2
PD1	4G1	PD1_4G1	devil	mouse	yes/no. doesn't block PD-1-His 20150701 to PDL1, blocked PD-1-Fc on 20151105 to PDL1 and PDL2
PD1	5A6	PD1_5A6	devil	mouse	no. Does not block PD1-His to PDL1 20150701 and PD1-Fc to PDL1 and PDL2 20151105
PD1	5F11	PD1_5F11	devil	mouse	yes/no, doesn't block PD-1-His to PDL2 20150701, blocked PD-1-Fc to PDL1 PDL220151105
PD1	7G12	PD1_7G12	devil	mouse	partial. Blocks PD1-Fc to PDL1 20151105 but not PD1-Fc to PDL2 20151105
PD1	8E1	PD1_8E1	devil	mouse	partial PD1-His to PDL1 block 20150701 and PD1-Fc to PDL1 20151105, but does not block PDL2 20151105

Supplementary Table 3. Summary of histological slides and results.

individual	antigen	clone	antibody_ID	TD_num	TD_name	tissue	tumor	slide	result	positive	strain	sex	age	captive_wild
1	PD-1	3G8	PD-1_3G8	181	Missy	tum	1	TD_181_Missy_tum	negative	0	DFT1	F	1	captive
1	PD-L1	1F8	PD-L1_1F8	181	Missy	tum	1	TD_181_Missy_tum	negative	0	DFT1	F	1	captive
2	PD-L1	1F8	PD-L1_1F8	269	_	tum	1	TD_269___tum	negative	0	DFT1	F	NA	wild
3	PD-1	3G8	PD-1_3G8	307	_	tum	1	TD_307___tum	negative	0	DFT1	F	1	wild
3	PD-L1	1F8	PD-L1_1F8	307	_	tum	1	TD_307___tum	1-3 strong PD-L1+ cells in the tu	1	DFT1	F	1	wild
4	PD-1	3G8	PD-1_3G8	316	Lottie	spleen-LN	0	TD_316_Lottie_spleen-LN	NA	NA	DFT1	NA	NA	captive
4	PD-1	3G8	PD-1_3G8	316	Lottie	LHS-tum	1	TD_316_Lottie_LHS-tum	negative	0	DFT1	NA	NA	captive
4	PD-L1	1F8	PD-L1_1F8	316	Lottie	spleen-LN	0	TD_316_Lottie_spleen-LN	Low numbers of lymphocytes wit	1	DFT1	NA	NA	captive
4	PD-L1	1F8	PD-L1_1F8	316	Lottie	LHS-tum	1	TD_316_Lottie_LHS-tum	negative	0	DFT1	NA	NA	captive
5	PD-1	3G8	PD-1_3G8	318	Elsie	tum-29.4.13	1	TD_318_Elsie_tum-29.4.13	NA	NA	DFT1	NA	NA	captive
5	PD-L1	1F8	PD-L1_1F8	318	Elsie	tum-29.4.13	1	TD_318_Elsie_tum-29.4.13	> 20 PD-L1+ between large tum	1	DFT1	NA	NA	captive
6	PD-1	3G8	PD-1_3G8	374	_	spleen-LN	0	TD_374___spleen-LN	Low numbers of lymphocytes wit	NA	DFT1	NA	NA	captive
6	PD-L1	1F8	PD-L1_1F8	374	_	spleen-LN	0	TD_374___spleen-LN	PD-L1+ cells throughout LN	1	DFT1	NA	NA	captive
7	PD-1	3G8	PD-1_3G8	467	RV	spleen-LN	0	TD_467_RV_spleen-LN	strong PD-1+ cells mostly cortica	1	DFT2	M	3.5	wild
7	PD-1	3G8	PD-1_3G8	467	RV	kidney-met	1	TD_467_RV_kidney-met	DFT2 metastasis to renal cortex.	0	DFT2	M	3.5	wild
7	PD-1	3G8	PD-1_3G8	467	RV	lung-met	1	TD_467_RV_lung-met	Low numbers of lymphocytes wit	1	DFT2	M	3.5	wild
7	PD-1	3G8	PD-1_3G8	467	RV	tum1A	1	TD_467_RV_tum1A	negative	0	DFT2	M	3.5	wild
7	PD-1	3G8	PD-1_3G8	467	RV	tum1B	1	TD_467_RV_tum1B	NA	NA	DFT2	M	3.5	wild
7	PD-L1	1F8	PD-L1_1F8	467	RV	spleen-LN	0	TD_467_RV_spleen-LN	patchy PD-L1+ cells	1	DFT2	M	3.5	wild
7	PD-L1	1F8	PD-L1_1F8	467	RV	kidney-met	1	TD_467_RV_kidney-met	negative	0	DFT2	M	3.5	wild
7	PD-L1	1F8	PD-L1_1F8	467	RV	lung-met	1	TD_467_RV_lung-met	10-20 scattered PD-L1+ cells no	1	DFT2	M	3.5	wild
7	PD-L1	1F8	PD-L1_1F8	467	RV	tum1A	1	TD_467_RV_tum1A	negative	0	DFT2	M	3.5	wild
7	PD-L1	1F8	PD-L1_1F8	467	RV	tum1B	1	TD_467_RV_tum1B	negative	0	DFT2	M	3.5	wild
8	PD-1	3G8	PD-1_3G8	500	_	T2	1	TD_500___T2	PD-1+ cells, but no PD-1+ in lar	1	DFT2	M	4	wild
8	PD-L1	1F8	PD-L1_1F8	500	_	T2	1	TD_500___T2	Low numbers of plasma cell like	1	DFT2	M	4	wild
9	PD-L1	1F8	PD-L1_1F8	504	_	T9	1	TD_504___T9	>20 PD-L1+ cells in glands near	1	DFT1	F	1	wild
10	PD-L1	1F8	PD-L1_1F8	505	_	LN	0	TD_505___LN	PD-L1+ cells throughout LN	1	DFT1	F	3	wild
10	PD-L1	1F8	PD-L1_1F8	505	_	lung	0	TD_505___lung	single PD-L1+ cell in large tumo	1	DFT1	F	3	wild
10	PD-L1	1F8	PD-L1_1F8	505	_	T1	1	TD_505___T1	3-10 PD-L1+ cells. Near gap in	1	DFT1	F	3	wild
10	PD-L1	1F8	PD-L1_1F8	505	_	T2	1	TD_505___T2	PD-L1+ cells present	1	DFT1	F	3	wild
10	PD-L1	1F8	PD-L1_1F8	505	_	T3	1	TD_505___T3	negative, mostly skin tissue in th	0	DFT1	F	3	wild
11	PD-L1	1F8	PD-L1_1F8	506	_	T1	1	TD_506___T1	negative	0	DFT1	F	1	wild
12	PD-L1	1F8	PD-L1_1F8	512	_	LN-met	0	TD_512___LN-met	There are low numbers of lymph	1	DFT1	F	2-3	wild
12	PD-L1	1F8	PD-L1_1F8	512	_	kidney-met	1	TD_512___kidney-met	Low numbers of plasma cells an	1	DFT1	F	2-3	wild
12	PD-L1	1F8	PD-L1_1F8	512	_	lung-met	1	TD_512___lung-met	patchy PD-L1+ cells near small t	1	DFT1	F	2-3	wild
12	PD-L1	1F8	PD-L1_1F8	512	_	T1	1	TD_512___T1	negative	0	DFT1	F	2-3	wild
12	PD-L1	1F8	PD-L1_1F8	512	_	T2	1	TD_512___T2	strong PD-L1++ in stroma, but c	1	DFT1	F	2-3	wild
13	PD-1	3G8	PD-1_3G8	514	_	T1	1	TD_514___T1	3 PD-1+ cells near a PD-L1+ cel	1	DFT1	NA	NA	wild
13	PD-1	3G8	PD-1_3G8	514	_	T2	1	TD_514___T2	negative	0	DFT1	NA	NA	wild
13	PD-L1	1F8	PD-L1_1F8	514	_	lung-met	1	TD_514___lung-met	4 PD-L1+ in tumor mass, 4 PD-L	1	DFT1	NA	NA	wild
13	PD-L1	1F8	PD-L1_1F8	514	_	T1	1	TD_514___T1	3 PD-L1+ cells in stroma near b	1	DFT1	NA	NA	wild
13	PD-L1	1F8	PD-L1_1F8	514	_	T2	1	TD_514___T2	negative	0	DFT1	NA	NA	wild
14	PD-1	3G8	PD-1_3G8	517	Merrick	spleen-LN	0	TD_517_Merrick_spleen-LN	PD-1+ in cortical gernimal cente	1	DFT1	M	4	captive
14	PD-1	3G8	PD-1_3G8	517	Merrick	T1	1	TD_517_Merrick_T1	negative	0	DFT1	M	4	captive
14	PD-L1	1F8	PD-L1_1F8	517	Merrick	spleen-LN	0	TD_517_Merrick_spleen-LN	Less PD-L1+ cells than most LN	1	DFT1	M	4	captive
14	PD-L1	1F8	PD-L1_1F8	517	Merrick	T1	1	TD_517_Merrick_T1	negative	0	DFT1	M	4	captive
15	PD-1	3G8	PD-1_3G8	518	Tip	LHS-mass	1	TD_518_Tip_LHS-mass	negative	0	DFT1	M	7	captive
15	PD-L1	1F8	PD-L1_1F8	518	Tip	LHS-mass	1	TD_518_Tip_LHS-mass	few PD-L1+ cells	1	DFT1	M	7	captive
16	PD-1	3G8	PD-1_3G8	546	Stinky	Kidney	0	TD_546_Stinky_Kidney	negative	0	DFT1	NA	NA	captive
16	PD-1	3G8	PD-1_3G8	546	Stinky	LN-A	0	TD_546_Stinky_LN-A	strong PD-1+ section	1	DFT1	NA	NA	captive
16	PD-1	3G8	PD-1_3G8	546	Stinky	interscap	1	TD_546_Stinky_interscap	strong PD-1+	1	DFT1	F	NA	captive
16	PD-1	3G8	PD-1_3G8	546	Stinky	LHS-RumpA	1	TD_546_Stinky_LHS-RumpA	TILs are PD-1+ in tumor. PRX+	1	DFT1	NA	NA	captive

16	PD-1	3G8	PD-1_3G8	546	Stinky	LHS-RumpB	1	TD_546_Stinky_LHS-RumpB	NA	NA	DFT1	NA	NA	captive
16	PD-L1	1F8	PD-L1_1F8	546	Stinky	Kidney	0	TD_546_Stinky_Kidney	1 PD-L1+ cell in blood vessel	1	DFT1	NA	NA	captive
16	PD-L1	1F8	PD-L1_1F8	546	Stinky	LN-A	0	TD_546_Stinky_LN-A	PD-L1+ cells throughout LN	1	DFT1	NA	NA	captive
16	PD-L1	1F8	PD-L1_1F8	546	Stinky	Lung-B	0	TD_546_Stinky_Lung-B	1 PD-L1+ cell. Might be other po	1	DFT1	NA	NA	captive
16	PD-L1	1F8	PD-L1_1F8	546	Stinky	interscap	1	TD_546_Stinky_interscap	few PD-L1+ cells	1	DFT1	F	NA	captive
16	PD-L1	1F8	PD-L1_1F8	546	Stinky	LHS-RumpA	1	TD_546_Stinky_LHS-RumpA	few PD-L1+ cells	1	DFT1	NA	NA	captive
16	PD-L1	1F8	PD-L1_1F8	546	Stinky	LHS-RumpB	1	TD_546_Stinky_LHS-RumpB	4 PD-L1+ cells in tumour, but mi	1	DFT1	NA	NA	captive
16	PD-L1	1F8	PD-L1_1F8	546	Stinky	RHS-T1	1	TD_546_Stinky_RHS-T1	few PD-L1+ cells, but staining is	1	DFT1	F	NA	captive
16	PD-L1	1F8	PD-L1_1F8	546	Stinky	RHS-tum	1	TD_546_Stinky_RHS-tum	strong PD-L1++	1	DFT1	F	NA	captive
16	PD-L1	1F8	PD-L1_1F8	546	Stinky	Sh-B1	1	TD_546_Stinky_Sh-B1	few PD-L1+	1	DFT1	F	NA	captive
16	PD-L1	1F8	PD-L1_1F8	546	Stinky	Tum-interscap	1	TD_546_Stinky_Tum-interscap	negative	0	DFT1	NA	NA	captive
17	PD-1	3G8	PD-1_3G8	_	Oryx	tum	1	TD___Oryx_tum	~10 PD-1+ cells scattered in blo	1	DFT1	NA	NA	wild
17	PD-L1	1F8	PD-L1_1F8	_	Oryx	tum	1	TD___Oryx_tum	negative	0	DFT1	NA	NA	wild
18	PD-1	3G8	PD-1_3G8	_	Octavio	tum	1	TD___Octavio_tum	negative	0	DFT1	NA	NA	wild
18	PD-L1	1F8	PD-L1_1F8	_	Octavio	tum	1	TD___Octavio_tum	cluster of 5-10 PD-L1+ cells in bl	1	DFT1	NA	NA	wild
